# The start of the Austrian response to the COVID-19 crisis: a personal account

**DOI:** 10.1007/s00508-020-01693-y

**Published:** 2020-06-12

**Authors:** Markus Müller

**Affiliations:** Rector, Medical University of Vienna, Spitalgasse 23, 1090 Vienna, Austria

The first reports about human-to-human transmission of a new severe acute respiratory syndrome (SARS) virus in Wuhan, China, which emerged in January 2020 met a relaxed sentiment among experts and the public in Austria who were reminiscent of similar reports on SARS1, H1N1 or H5N1. Even warnings by the World Health Organization (WHO) of COVID-19 as a “health emergency of international concern” [1], media coverage on the spread of the virus in Asia and on the situation on cruise ship Diamond Princess [2] and first reports and preprints about clinical courses and disease characteristics [3–7] did not lead to any particular precautions or change in sentiment. It was widely assumed that containment of the disease would be accomplished in Asia like for SARS. Increasing knowledge about non-symptomatic spreading and high case fatality rates in the older population, led to comparisons with influenza and the European Centre for Disease Prevention and Control (ECDC) risk assessment fourth update reported a “low” risk of COVID-19 for European member states on 14 February 2020 [6].

After personal consultations with the Head of the Vienna City Hospitals, I requested a joint meeting with the Magistrate of the City of Vienna on the consequences of a potential COVID-19 threat in Vienna, which led to a first meeting of stakeholders in Vienna on 23 February and the implementation of a crisis board. Among other issues it concluded that (a) SARS-CoV‑2 test capacities, which had been established at the Institute of Virology at the Medical University of Vienna needed to be scaled up, (b) any new COVID-19 cases should first be contained at home and contact traced, (c) all community-acquired pneumonia (CAP) cases in Vienna hospitals should be screened for SARS-CoV‑2. On the same day, I made contact with the Austrian Government via a Parliamentarian of the Green Party, which was responsible for the Ministry of Health in a coalition with the Peoples Party. From an administrative point of view, Austria was in a vulnerable position, as the new government was in office only until January 2020 and the National Sanitary Council, whose term had expired on 31 December 2019, had not been re-established. The position of the National General Director for Public Health had been removed by a previous government and never been reinstalled and the pandemic plan dated back to the emergence of H5N1 and H1N1 and focused on influenza.

On 24 February I was asked by the Austrian Ministry of Health to suggest a panel of experts who could advise the Minister of Health. On 28 February this advisory council was introduced to the public and first pursued a COVID-19 containment strategy. The first case of COVID-19 in Austria was reported on 25 February and on 27 February the CAP screening initiative reported a first COVID-19 case in a Vienna Hospital intensive care unit (ICU). On 28 February the WHO published a report of the WHO-China joint mission on COVID-19 providing further details on the outbreak in China, including fatality rates and asymptomatic carriers [7].

At the beginning of March, the effective reproduction number (R_eff_) of COVID-19 infections in Austria was around 3.0, the doubling time around 2 days and the daily increase around 40%. It was clear from the beginning that the available data from China were probably prone to substantial reporting bias but despite the question of the individual risk profile, different modelling approaches showed that a synchronized appearance of severe cases would probably also overwhelm the Austrian capacities, which was in line with a report from the Imperial College [8]. An unanswered question was an “iceberg” of asymptomatic infections. which was reported to range from 1% to 40% or even 86% [2, 9–12]. In line with reports on a destabilizing situation in northern Italy [13, 14] and a cluster of COVID-19 cases in skiing resorts in Tyrol it became clear that SARS-CoV‑2 had reached the level of community transmission. It was therefore advised to switch from containment to mitigation, level 2 according to the ECDC RRP5 (Rapid risk assessment – Outbreak of novel coronavirus disease 2019 [COVID-19]: increased transmission globally – fifth update) [15], and to focus on social distancing similarly to the Swiss approach.

On 10 March the Medical University implemented mandatory distance learning for all students to protect its large teaching hospital, the Vienna General Hospital. On the same day, the government and the Parliament unanimously implemented the first steps of a number of measures, which led to a ban of public events, travel restriction closing of schools, universities, restaurants, most businesses on 16 March. Following the first announcement of measures in Austria, R_eff_ was reduced from above 3.0 to below 0.7, which enabled a gradual lifting of restrictions. From 14 April Austria, together with Denmark, was the first European country to lift first restrictions, at the beginning with the opening of shops, followed by restaurants and schools. Mandatory wearing of face masks in defined situations was introduced in line with emerging reports on likely effectiveness of masks [16, 17].

The Austrian strategy, which was legalized by Austrian COVID Act was initially supported unequivocally by all parliamentary parties and the public; however, a shift in public opinion could be observed from the beginning of April onwards, which was also seen in other countries worldwide. The initial UK, Dutch and US attempts to pursue herd immunity were never broadly considered in the public opinion, but alternative strategies were advocated, notably the softer Swedish approach and the crisis management was reproached for its strategy nationally but taken as a role model internationally [18–20]. A further study of the Imperial College estimated that the Austrian interventions had led to a reduction of fatalities by approximately 50% and estimated an infection rate in the population of 1.1% (0.36–3.1%), which was largely in line with later random samples [21].

First assessments of different national strategies to cope with SARS-CoV‑2 in the early phase noted that: “Germany and Austria stand out as nations that adopted aggressive and early control strategies compared with Italy, France and Spain, which implemented similar measures, including lockdown, but later in their epidemics”, and that, “so far, Germany and Austria have, per capita, seen a fraction of the deaths from COVID-19 of these other countries” [22].

## References

[1] World Health Organization. WHO timeline - COVID-19. 2020. www.who.int/news-room/detail/27-04-2020-who-timeline---covid-19.

[2] Mizumoto K, Kagaya K, Zarebski A, Chowell G (2020). Estimating the asymptomatic proportion of coronavirus disease 2019 (COVID-19) cases on board the Diamond Princess cruise ship, Yokohama, Japan. Euro Surveill.

[3] Yang X, Yu Y, Xu J, Shu H, Xia J, Liu H, Wu Y, Zhang L, Yu Z, Fang M, Yu T, Wang Y, Pan S, Zou X, Yuan S, Shang Y (2020). Clinical course and outcomes of critically ill patients with SARS-CoV-2 pneumonia in Wuhan, China: a single-centered, retrospective, observational study. Lancet Respir Med.

[4] Zhao XY, Xu XX, Yin HS, Hu QM, Xiong T, Tang YY, Yang AY, Yu BP, Huang ZP (2020). Clinical characteristics of patients with 2019 coronavirus disease in a non-Wuhan area of Hubei Province, China: a retrospective study. BMC Infect Dis.

[5] Guan WJ, Ni ZY, Hu Y, Liang WH, Ou CQ, He JX, Liu L, Shan H, Lei CL, Hui DSC, Du B, Li LJ, Zeng G, Yuen KY, Chen RC, Tang CL, Wang T, Chen PY, Xiang J, Li SY, Wang JL, Liang ZJ, Peng YX, Wei L, Liu Y, Hu YH, Peng P, Wang JM, Liu JY, Chen Z, Li G, Zheng ZJ, Qiu SQ, Luo J, Ye CJ, Zhu SY, Zhong NS (2020). China medical treatment expert group for Covid-19. Clinical characteristics of coronavirus disease 2019 in China. N Engl J Med.

[6] European Centre for Disease Prevention and Control. Risk assessment: outbreak of severe acute respiratory syndrome coronavirus 2 (SARS-CoV-2): increased transmission beyond China – fourth update. 2020. www.ecdc.europa.eu/en/publications-data/outbreak-severe-acute-respiratory-syndrome-coronavirus-2-sars-cov-2-increased.

[7] World Health Organization. Report of the WHO-China joint mission on coronavirus disease 2019 (COVID-19). 2020. www.who.int/publications-detail/report-of-the-who-china-joint-mission-on-coronavirus-disease-2019-(covid-19).

[8] Ferguson NM, Laydon D, Nedjati-Gilani G, et al. Report 9 - Impact of non-pharmaceutical interventions (NPIs) to reduce COVID-19 mortality and healthcare demand. 2020. www.imperial.ac.uk/mrc-global-infectious-disease-analysis/covid-19/report-9-impact-of-npis-on-covid-19/. 10.1007/s11538-020-00726-x PMC7140590 32270376

[9] Wu JT, Leung K, Bushman M, Kishore N, Niehus R, de Salazar PM, Cowling BJ, Lipsitch M, Leung GM (2020). Estimating clinical severity of COVID-19 from the transmission dynamics in Wuhan, China. Nat Med.

[10] Wu Z, McGoogan JM (2020). Characteristics of and important lessons from the coronavirus disease 2019 (COVID-19) outbreak in China: summary of a report of 72 314 cases from the Chinese center for disease control and prevention. JAMA.

[11] Nishiura H, Kobayashi T, Miyama T, Suzuki A, Jung S, Hayashi K, Kinoshita R, Yang Y, Yuan B, Akhmetzhanov AR, Linton NM (2020). Estimation of the asymptomatic ratio of novel coronavirus infections (COVID-19). Int J Infect Dis.

[12] Li R, Pei S, Chen B, Song Y, Zhang T, Yang W, Shaman J (2020). Substantial undocumented infection facilitates the rapid dissemination of novel coronavirus (SARS-CoV2). Science.

[13] Remuzzi A, Remuzzi G (2020). COVID-19 and Italy: what next?. Lancet.

[14] Onder G, Rezza G, Brusaferro S (2020). Case-fatality rate and characteristics of patients dying in relation to COVID-19 in Italy. JAMA.

[15] European Centre for Disease Prevention and Control. Rapid risk assessment: outbreak of novel coronavirus disease 2019 (COVID-19): increased transmission globally – fifth update. 2020. www.ecdc.europa.eu/en/publications-data/rapid-risk-assessment-outbreak-novel-coronavirus-disease-2019-covid-19-increased. 10.2807/1560-7917.ES.2020.25.9.2003051 PMC7068166 32156331

[16] Leung NHL, Chu DKW, Shiu EYC, Chan KH, McDevitt JJ, Hau BJP, Yen HL, Li Y, Ip DKM, Peiris JSM, Seto WH, Leung GM, Milton DK, Cowling BJ (2020). Respiratory virus shedding in exhaled breath and efficacy of face masks. Nat Med.

[17] Stadnytskyi V, Bax CE, Bax A, Anfinrud P (2020). The airborne lifetime of small speech droplets and their potential importance in SARS-CoV-2 transmission. Proc Natl Acad Sci U S A.

[18] Kupferschmidt K (2020). The lockdowns worked—but what comes next?. Science.

[19] Wingrove J, Groendahl B, Kong K. White House looks to Austria, South Korea on reopening ideas. 2020. https://www.bloomberg.com/news/articles/2020-05-10/white-house-looks-to-austria-south-korea-for-reopening-ideas.

[20] Pancevski B. Countries that kept a lid on coronavirus look to each other to revive their economies. 2020. https://www.wsj.com/articles/countries-that-kept-a-lid-on-coronavirus-look-to-each-other-to-revive-their-economies-11588424855.

[21] Flaxman S, Mishra S, Gandy A, et al. Report 13 - Estimating the number of infections and the impact of non-pharmaceutical interventions on COVID-19 in 11 European countries. 2020. http://www.imperial.ac.uk/mrc-global-infectious-disease-analysis/covid-19/report-13-europe-npi-impact/.

[22] Gibney E. Whose coronavirus strategy worked best? Scientists hunt most effective policies. Nature 2020;581, 15–16. 10.1038/d41586-020-01248-1 32341558

